# Facilitated Unidirectional Electron Transmission by Ru Nano Particulars Distribution on MXene Mo_2_C@g-C_3_N_4_ Heterostructures for Enhanced Photocatalytic H_2_ Evolution

**DOI:** 10.3390/molecules29071684

**Published:** 2024-04-08

**Authors:** Qiuyu Chen, Zonghan Huang, Meng Liu, Xiaoping Li, Yuxuan Du, Xiaobao Chen, Dahu Ding, Shengjiong Yang, Yang Chen, Rongzhi Chen

**Affiliations:** 1 College of Resources and Environment, University of Chinese Academy of Sciences, Beijing 100049, China; chenqiuyu20@mails.ucas.ac.cn (Q.C.);; 2 Yanshan Earth Critical Zone and Surface Fluxes Research Station, University of Chinese Academy of Sciences, Beijing 100049, China; 3 College of Resources and Environmental Sciences, Nanjing Agricultural University, Nanjing 210095, China; 4 Key Laboratory of Environmental Engineering, Xi’an University of Architecture and Technology, No. 13, Yanta Road, Xi’an 710055, China

**Keywords:** metal nanoparticles, MXene Mo_2_C, graphite carbon nitride, unidirectional electronic transmission, hydrogen evolution

## Abstract

Precious metals exhibit promising potential for the hydrogen evolution reaction (HER), but their limited abundance restricts widespread utilization. Loading precious metal nanoparticles (NPs) on 2D/2D heterojunctions has garnered considerable interest since it saves precious metal consumption and facilitates unidirectional electron transmission from semiconductors to active sites. In this study, Ru NPs loaded on MXenes Mo_2_C by an in-site simple strategy and then formed 2D/2D heterojunctions with 2D g-C_3_N_4_ (CN) via electrostatic self-assembly were used to enhance photocatalytic H_2_ evolution. Evident from energy band structure analyses such as UV-vis and TRPL, trace amounts of Ru NPs as active sites significantly improve the efficiency of the hydrogen evolution reaction. More interestingly, MXene Mo_2_C, as substrates for supporting Ru NPs, enriches photoexcited electrons from CN, thereby enhancing the unidirectional electron transmission. As a result, the combination of Ru-Mo_2_C and CN constructs a composite heterojunction (Ru-Mo_2_C@CN) that shows an improved H_2_ production rate at 1776.4 μmol∙g^−1^∙h^−1^ (AQE 3.58% at 400 nm), which is facilitated by the unidirectional photogenerated electron transmission from the valence band on CN to the active sites on Ru (CN→Mo_2_C→Ru). The study offers fresh perspectives on accelerated unidirectional photogenerated electron transmission and saved precious metal usage in photocatalytic systems.

## 1. Introduction

The use of semiconductor photocatalysis for hydrogen generation is a hopeful solution for the issues of energy scarcity and pollution [1,2,3]. However, the efficiency of electronic-hole pair separation and reduction reaction kinetics in surface water are crucial issues that impact photocatalytic hydrogen production [4,5,6]. Various techniques, such as morphology modulation, valence band potential engineering, and heterogeneous structure building, have been utilized to address the aforementioned key problems [7,8,9,10]. Precious metals, notably platinum (Pt), are recognized for their efficacy as co-catalysts to form heterojunctions with semiconductors by lowering the overpotentials required for surface water reduction reactions and enhancing charge transmission [11]. Nevertheless, the limited availability and elevated costs hinder the widespread application of precious metals as co-catalysts [12,13]. Hence, there has been a growing focus in recent years on minimizing the amount of precious metal co-catalysts used in photocatalytic hydrogen generation [14,15].

Ruthenium (Ru), a relatively cost-effective metal from the platinum group, has Ru-H bond strengths comparable to those of Pt-H bonds, which suggests that it could be a viable substitute for Pt [16,17]. To save on the use of precious metals, many studies have attempted to load Ru nanoparticles (NPs) onto other co-catalysts [18,19]. Ru NPs can change the electronic structure of heterojunctions and introduce abundant active sites, resulting in several times the hydrogen production performance of the original catalyst [20,21]. Incorporating Ru NPs onto two-dimensional (2D) structures, such as g-C_3_N_4_, is more achievable for the uniform distribution of active sites due to the extensive expanse of surface area [22,23].

2D g-C_3_N_4_ is an ideal semiconductor photocatalyst because of its stable physicochemical properties and suitable band gap [24]. Nevertheless, the extensive use of pure g-C_3_N_4_ in photocatalysis is limited due to its slow charge transfer rate and rapid complexation of photoexcited electron-hole pairs [25]. To address the mentioned issues, MXene Mo_2_C with superior conductivity can create a 2D/2D heterostructure with g-C_3_N_4_ [26], with chemical bonds and van der Waals force accelerating electron migration, thus greatly facilitating the catalytic reaction [27]. Furthermore, Mo_2_C exhibits excellent metal-like conductivity and a lower valence band position to form a Schottky junction with CN, thereby promoting the directional migration of photogenerated electrons via the junction [28]. The metalloid’s conductivity can be attributed to its Pt-like d-orbital structure, which is created by hybridizing Mo d-orbitals with C s/p-orbitals [29]. However, the higher bond energy of the Mo-H bond (65–75 kcal mol^−1^) in Mo_2_C MXene results in robust adsorption of H^+^ ions, hence exhibiting limited desorption of H_2_ molecules from the Mo atoms [30,31]. Therefore, it is essential to incorporate hydrogen evolution reaction (HER) activity into Mo_2_C to establish a balance in atomic hydrogen adsorption and desorption for improving photocatalytic hydrogen production [32].

This research combines a Ru-doped 2D MXene Mo_2_C co-catalyst with 2D g-C_3_N_4_ layers to improve their hydrogen evolution efficiency. The conductive Mo_2_C enriches the photogenerated electrons generated in CN through the Schottky junction, as evidenced by time-resolved photoluminescence (TRPL) and electrochemical impedance spectroscopy (EIS). In addition, based on energy band structure analysis and the UV-Vis absorbance spectrum (UV-Vis), the active sites introduced by Ru NPs at the interface enhance the surface water reduction reaction, thereby guiding the enriched electrons to Ru. The Schottky barriers between Mo_2_C and CN inhibit the reverse flow and recombination of electron-hole pairs, thus facilitating unidirectional electron transmission from the semiconductor to the active sites (CN → Mo_2_C → Ru). As anticipated, the Ru-Mo_2_C@g-C_3_N_4_ heterostructure demonstrated a notable hydrogen production rate of 1776.4 μmol∙g^−1^∙h^−1^ and an apparent quantum efficiency (AQE) of 3.58% at 400 nm, surpassing the majority of reported Mo_2_C/g-C_3_N_4_ photocatalytic systems. This study presents a strategy for unidirectional electron migration by loading trace precious metal NPs, which provides guidance for improving charge migration efficiency in photocatalytic hydrogen production.

## 2. Results and Discussion

### 2.1. Construction Strategy of Ru-Mo_2_C@CN

**Figure 1:**
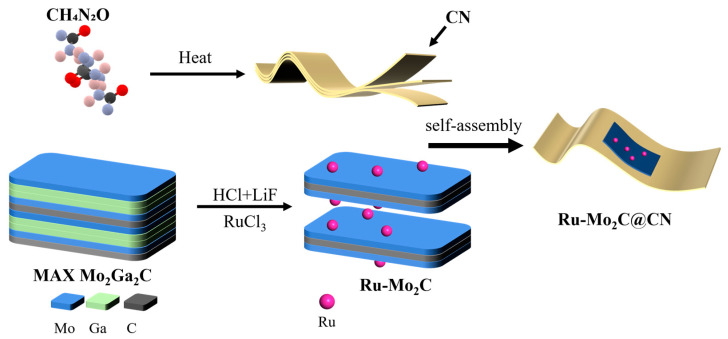
Simplified synthesis diagram of Ru-Mo_2_C@CN.

The production of Ru-Mo_2_C@CN photocatalysts followed the specific procedure outlined in Figure 1. Briefly, 2D MXene Mo_2_C was prepared by etching Mo_2_Ga_2_C with HCl-LiF in hydrothermal conditions, while Ru/Mo_2_C was fabricated by etching Mo_2_Ga_2_C with HCl-LiF-RuCl_3_. In the second step, Ru^3+^ in the solution is transformed into Ru clusters attached to the Mo_2_C surface, with Ga being oxidized and subsequently eliminated. This redox reaction occurs spontaneously from a thermodynamic perspective. The Ru-Mo_2_C@CN was eventually formed via the electrostatic self-assembly of 2D g-C_3_N_4_ nanosheets and MXene Ru-Mo_2_C layers.

### 2.2. Synthesis and Structural Morphology of Ru-Mo_2_C@CN

**Figure 2:**
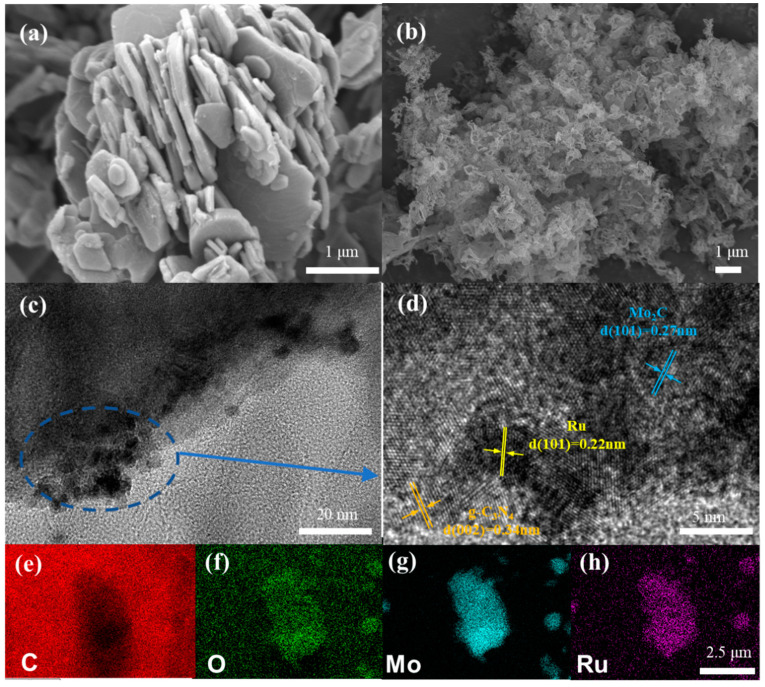
Analysis of the microstructure and morphology of Ru-Mo_2_C. (**a**) Ru-Mo_2_C SEM image; (**b**) 2D structure of CN SEM image; (**c**,**d**) HRTEM images; (**e**–**h**) EDX elemental mappings.

The MXene Mo_2_C without Ru loading exhibits a typical layered structure, which will help prevent the aggregation of Ru nanoparticles (Figure S1). The scanning electron microscope (SEM) images (Figure 2a,b) displayed that the Ru-Mo_2_C nanosheets showed a flat and smooth 2D structure after etching, while the CN nanosheets showed a reticulated 2D structure, which created the possibility of electrostatic self-assembly between the two. High-resolution transmission electron microscopy (HRTEM) revealed the surface morphology, indicating the existence of Ru NPs in Figure 2c,d. As shown in Figure 2c, Ru supported on the Mo_2_C surface consists of nanoparticles smaller than 5 nm, which provide more abundant catalytic active sites for photocatalytic reactions compared with Ru clusters. The HRTEM image in Figure S2 also proves this conclusion. From the HRTEM image of Figure 2d, the Ru-Mo_2_C@CN is assembled from Mo_2_C nanosheets loaded with Ru NDs and 2D g-C_3_N_4_ nanosheets. Furthermore, as shown in Figure 2e–h, energy dispersive X-ray spectroscopy (EDX) analysis indicated that Ru NPs are uniformly dispersed on the Mo_2_C surface. The above characterization indicates that MXene can serve as a carrier for the highly dispersed loading of precious metals. Even if the amount of precious metals added is small, they can uniformly adhere to the surface of MXene.

**Figure 3:**
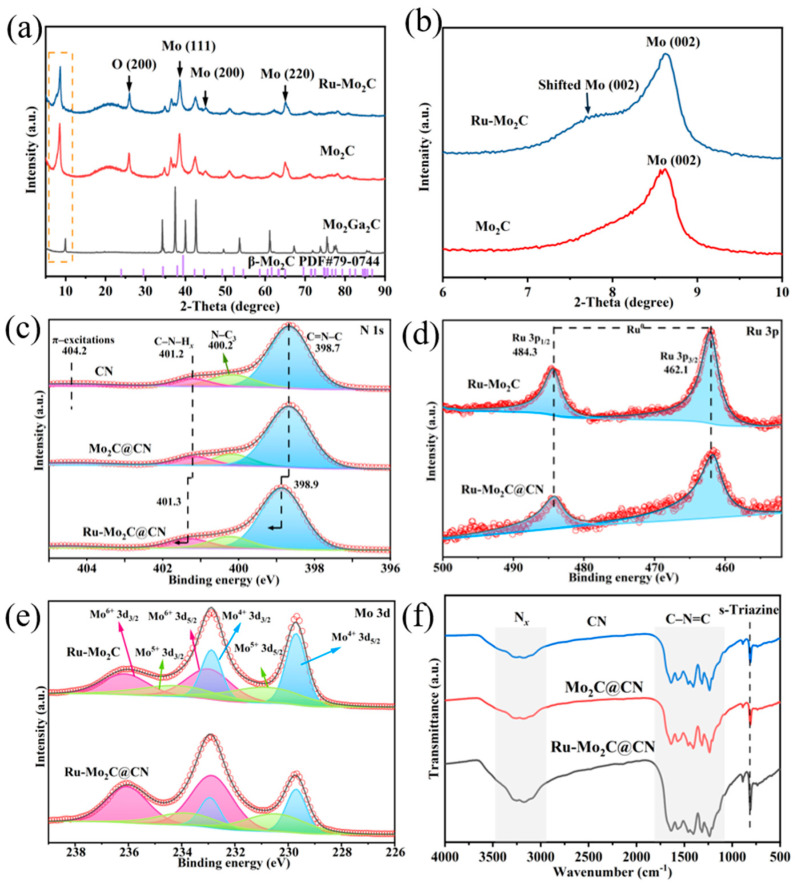
Analysis of Ru-Mo_2_C through structural and spectroscopic characterization. (**a**) XRD patterns of Mo_2_Ga_2_C, Mo_2_C, and Ru-Mo_2_C. (**b**) Enlarged Mo (002) diffraction peaks. High-resolution XPS spectra of (**c**) N 1s, (**d**) Ru 3p, and (**e**) Mo 3d. (**f**) FTIR spectra of CN, Mo_2_C@CN, and Ru-Mo_2_C@CN.

The absence of typical metallic Ru phase peaks in the XRD scan of Mxene could be attributed to the limited presence of Ru content on the surface (Figure 3a), falling below the detection threshold, or because the Ru grains are too small on the surface of Mxene to be diffracted during the X-ray diffraction (XRD) scanning. In addition, the (002) diffraction peak of Mo at 8.6° is somewhat shifted due to the introduction of Ru [33]. The shifted peaks showed that Ru clusters disrupt the integrity of the lattice. X-ray photoelectron spectrometer (XPS) analysis provides additional details on the surface chemical properties of the Ru-Mo_2_C@CN photocatalyst. The N 1s spectra display three distinct peaks at 401.9, 400.2, and 398.7 eV, corresponding to terminal amino groups (C-N-H_x_), tertiary nitrogen (N-C_3_), and sp2-hybridized nitrogen (C=N-C), as described in references [34,35]. Ru-Mo_2_C@CN shows a positive shift in the typical peaks of the N spectra compared to CN, suggesting unidirectional electron transmission from CN to Ru-Mo_2_C.

This finding indicates that the close connections between Mo_2_C and CN could offer a successful route for electron transfer. Surprisingly, the XPS data (Table S1) showed that Ru NPs accounted for only 0.5% of the total surface elements in the Ru-Mo_2_C@CN, indicating that trace amounts of NPs can significantly reduce the dependence of HER on noble metals. The peaks at 484.3 and 462.1 eV in the Ru 3p spectra of Ru-Mo_2_C and Ru-Mo_2_C@CN are indicative of the Ru 3p_3/2_ and 3p_1/2_ associated with metallic Ru^0^ [36,37], indicating the successful Ru NPs deposition on Mo_2_C (Figure 3d and Figure S3). The Mo 3d spectrum of the Ru-Mo_2_C@CN sample (Figure 3e) can be deconvoluted into six peaks, which confirms the presence of Mo^4+^, Mo^5+^, and Mo^6+^ species. The peaks at 229.7 (3d_5/2_) and 232.9 eV (3d_3/2_) are attributed to Mo^4+^ ions, and the peaks observed at 233.0 (3d_5/2_) and 236.2 (3d_3/2_) belong to Mo^6+^ ions [38,39], due to the partial oxidation of Mo_2_C. Additionally, the sub-oxide Mo^5+^ peaks, which appear at 230.9 (3d_5/2_) and 234.0 eV (3d_3/2_), may contribute to the X-ray reduction of Mo^6+^ in XPS tests [40,41]. More importantly, the Mo 3D spectrum of Ru-Mo_2_C@CN exhibits a significant negative shift in comparison to the XPS spectra of the original Ru-Mo_2_C. The noticeable change is due to the successful interfacial interaction between the Mxene Mo_2_C and the CN nanosheets, resulting in a decrease in the bond strength of Mo-H in Ru-Mo_2_C@CN and boosting HER performance [42,43].

The Fourier transform infrared spectroscopy (FTIR) of Figure 3f suggests that both Mo_2_C@CN and Ru-Mo_2_C@CN photocatalysts exhibit comparable characteristic peaks to CN. Two separate peaks around 806 and 884 cm^−1^ are likely caused by the vibrational modes related to the triazine units’ breathing. Previous studies support the conclusion that triazine units are the primary molecular composition of CN materials, making this observation highly convincing [44]. A broadband was observed in the 1233–1637 cm^−1^ region, which can be attributed to CN heterocyclic stretching vibration. In addition, the peaks at approximately 1243, 1330, 1412, 1464, 1572, and 1635 cm^−1^ are indicative of the presence of C-N bonds and C=N bond structures in the catalyst [44,45]. A group of high points can be seen in the spectrum between 3068 and 3524 cm^−1^, likely caused by the absorption of water molecules and subsequent stretching vibrations of N–H bonds [46]. The appearance of spectral characteristic peaks of g-C_3_N_4_ in the above-mentioned composite materials confirms that Mo_2_C doping does not damage the structure of CN.

### 2.3. Photocatalytic Performance

**Figure 4:**
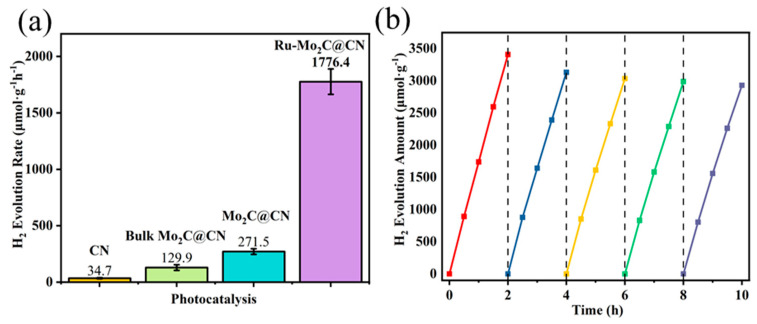
(**a**) The H_2_ evolution efficiency of all photocatalysts; (**b**) stability test of Ru-Mo_2_C@CN.

As shown in Figure 4a, the rapid rate of electron-hole pair complexation in CN leads to a low rate of H_2_ production through photocatalysis, with only minimal amounts of H_2_ detectable after 2 h of illumination. The combination of Mo_2_C and CN significantly increases the H_2_ production rate to 129.9 μmol∙g^−1^∙h^−1^ by providing electron transfer pathways and reducing the recombination of photo-generated electron-hole pairs. The increase in hydrogen production rate indicates that Mo_2_C can serve as a co-catalyst to improve the efficiency of H_2_ production in CN. Due to the large particle size of the bulk Mo_2_C, it could not be fully combined with CN. Therefore, a two-dimensional structure of Mxene Mo_2_C was chosen to bind with CN in this study. Substituting Mxene Mo_2_C for the original Mo_2_C in the composite material resulted in a renewed increase in the rate of photocatalytic H_2_ production to 271.5 μmol∙g^−1^∙h^−1^, demonstrating that the 2D/2D pairing effectively enhanced the movement of photogenerated carriers, leading to a decrease in carrier recombination rate. It is common knowledge that Ru has emerged as a viable alternative to Pt for generating hydrogen through photocatalysis. The photocatalytic H_2_ production performance of composite materials was further enhanced by doping 2D Mo_2_C with Ru. After introducing 0.5% Ru into Mo_2_C@CN, the photocatalysts reached a hydrogen production rate of 1776.4 μmol∙g^−1^∙h^−1^ and exhibited an apparent quantum yield (AQE) of 3.58% at 400 nm (detailed calculations in attachment), surpassing the performance of most reported Mo_2_C/g-C_3_N_4_ photocatalysts (Table S2). Importantly, to determine the successful construction of the heterojunction, we simply milled 10 mg of Ru-Mo_2_C with 90 mg of CN to obtain a physically mixed sample labeled Ru-Mo_2_C + CN. It was found that the hydrogen production rate of Ru-Mo_2_C + CN was much lower than that of Ru-Mo_2_C@CN, which can also be taken as evidence that the Ru-Mo2C@CN heterojunction was successfully constructed via electrostatic self-assembly (Figure S4). This excellent performance indicates that the 2D structure of Mo_2_C can serve as a good co-catalyst to improve the migration rate of photogenerated carriers, thereby further reducing the carrier complexation rate. In addition, the 2D structure of Mo_2_C can serve as a good intermediate carrier, providing more coverage areas for Ru as an active site.

Furthermore, it is crucial to consider the durability of the photocatalytic process, as demonstrated in Figure 4b, which illustrates the hydrogen generation rate by Ru-Mo_2_C@CN during a 10-h hydrogen production cycle. The photocatalyst showed good recyclability, as its hydrogen production rate decreased by less than 20% after five cycles. As shown in Figure S5, XRD images of the Ru-Mo_2_C@CN photocatalyst remain consistent before and after five cycles, indicating its good stability.

### 2.4. Photocatalytic Mechanism Analysis

**Figure 5:**
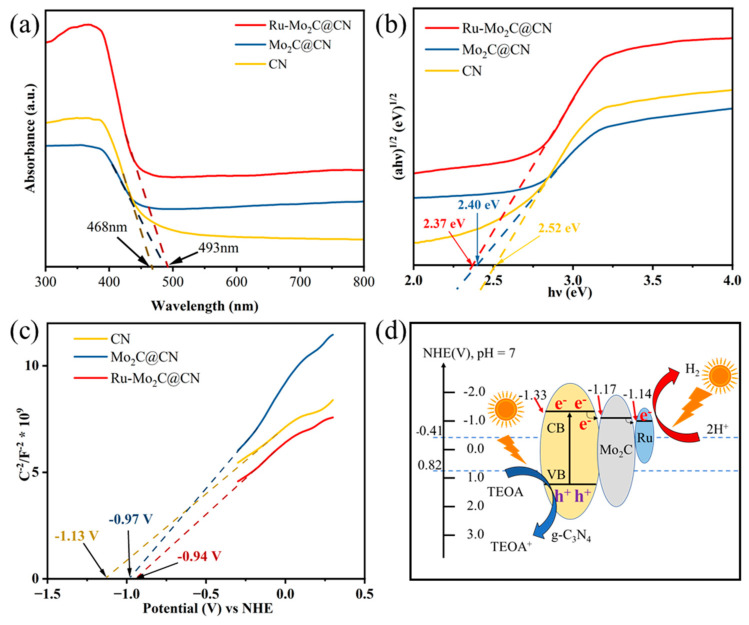
(**a**) UV-vis absorbance spectrum of CN, Mo_2_C@CN, and Ru-Mo_2_C@CN photocatalysts. (**b**) Tauc plots of three photocatalysts. (**c**) Mott–Schottky (M-S) curves of three photocatalysts (200 Hz). (**d**) Band structure schematic of the Ru-Mo_2_C@CN composite.

To understand why the photocatalytic performance improved, the light absorption abilities of various photocatalysts were initially examined using UV-Vis. As shown in Figure 5a, the intrinsic absorption edges of Ru-Mo_2_C@CN redshift to 493 nm, compared to the original CN’s inherent absorption edge at 468 nm. The redshift phenomenon is due to the strong visible light absorption ability of the Mo_2_C co-catalyst, indicating that Mo_2_C can promote the unidirectional electronic transmission from CN to Ru-Mo_2_C. Compared with Mo_2_C@CN, the absorbance of the photocatalyst can be slightly improved in the case of Ru-Mo_2_C doping, which indicates that Ru NPs as a co-catalyst can enhance the light-harvesting ability of composite materials. In addition, the overall absorbance of Ru-Mo_2_C@CN showed a significant improvement, which may be related to the influence of Ru clusters on the Mo lattice.

As shown in Figure 5b, Kubelka–Munk functional plots of three photocatalysts were obtained based on the UV-visible photometric spectral transformation. The bandgap value of Ru-Mo_2_C@CN (2.37 eV) is slightly lower than that of Mo_2_C@CN (2.40 eV) and much lower than that of CN (2.52 eV), which demonstrates the formation of a Schottky junction between Mo_2_C and CN, leading to a decrease in the bandgap. CN has a higher bandgap value of 2.52 eV compared to Mo_2_C@CN with 2.40 eV, whereas Ru-Mo_2_C@CN has a lower bandgap value of 2.37 eV. The difference in bandgap can be attributed to the close contact between Mo_2_C and CN, which generates Schottky junctions and decreases the bandgap of Mo_2_C@CN and Ru-Mo_2_C@CN. Furthermore, the reduction in the band gap suggests that the presence of a Mo_2_C co-catalyst enhances the ability of CN to be stimulated by visible light, aligning with the enhanced photocatalytic efficiency of Ru-Mo_2_C@CN.

To explore the effect of Mo_2_C on the semiconductor band gap, M-S measurements of the prepared catalyst were performed at a frequency of 200 Hz (Figure 5c). To find the flat band potential (E_FB_), extend the linear part of the M-S graph to the x-axis and locate the point where it intersects. In addition, the intercepts of the three catalysts in the M-S plot are all positive, indicating the characteristics of their n-type semiconductors [47]. Generally speaking, the conduction band potential (E_CB_) of n-type semiconductors is 0.2 eV greater than the E_FB_ [48]. Therefore, the E_CB_ for CN, Mo_2_C@CN, and Ru-Mo_2_C@CN concerning the Normal Hydrogen Electrode (NHE) are −1.33, −1.17, and −1.14 V, respectively. Based on the preceding results, Figure 5d illustrates the band structure of Ru-Mo_2_C@CN. When exposed to light, a large quantity of photoexcited electrons are produced within CN and jump into the conduction band (CB). Photoexcited electrons from CN are moved to Mo_2_C with a lower CB via the Schottky junction, then quickly transferred to Ru NPs for the reaction with H^+^ at the active site to produce hydrogen gas. The 0.16 V Schottky barriers between the CB of Mo_2_C and CN promote unidirectional electron migration.

**Figure 6:**
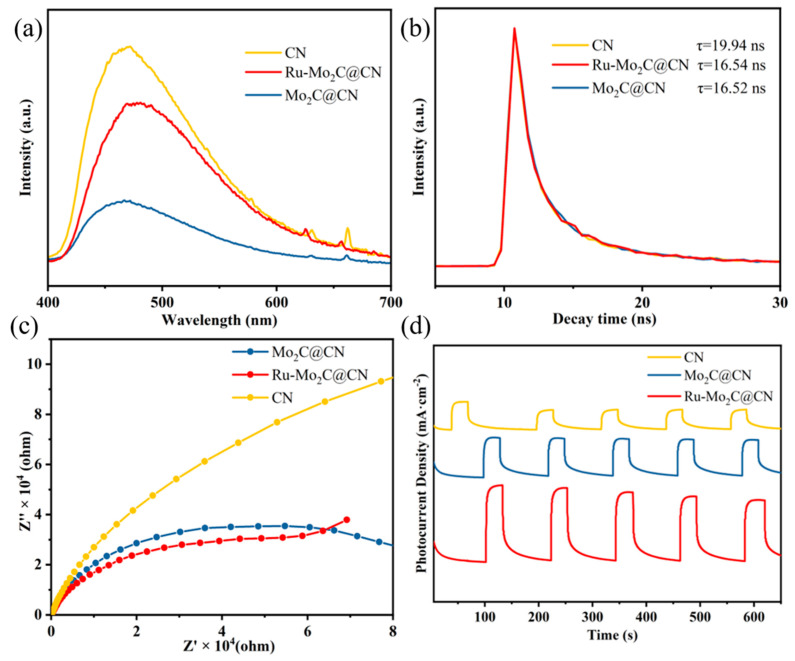
(**a**) PL and (**b**) TRPL spectra of three photocatalysts. (**c**) EIS Nyquist plots and (**d**) transient photocurrent plots of three photocatalysts.

To better understand the complexation process of photoexcited electron-hole pairs, photoluminescence (PL) spectra and TRPL spectra were used to analyze carriers’ behavior. Figure 6a shows the PL spectra of CN, Mo_2_C@CN, and Ru-Mo_2_C@CN. Due to electronic transitions between bands, each photocatalyst exhibits a distinct emission peak at approximately 470–490 nm under excitation at 325 nm, which is consistent with previous research results. Mo_2_C@CN exhibits lower PL intensity than CN, indicating successful suppression of electron-hole recombination through rapid charge transfer from CN to Mo_2_C. The Ru-Mo_2_C@CN composite shows a slightly elevated PL intensity compared to Mo_2_C@CN, accompanied by a significant peak redshift. This observation suggests that the Ru NPs may act as catalytic sites for HER, thereby enhancing the production rate of electron-hole pairs within the system, which is consistent with the results of UV-vis testing. The more electron-hole pairs are generated, the more electron-hole pairs are recombined, ultimately leading to higher PL intensity. As shown in Figure 6b, the TRPL spectra of the prepared catalyst all conform to double exponential fitting (detailed data in Table S3). Among them, τ_1_ denotes the duration of fluorescence decay for electrons transitioning from the conduction band to the valence band of CN, while τ_2_ denotes the duration of fluorescence decay for the recombination of electron-hole pairs generated by light on the CN. The average fluorescence lifetime of the TRPL spectra was calculated by the following equation [49]:

$$\tau =(A_{1}\tau_{1}^{2}+A_{2}\tau_{2}^{2})/(A_{1}\tau_{1}+A_{2}\tau_{2})$$

Following the addition of Mo_2_C co-catalysts, both τ_2_ and τ were significantly reduced, whereas the presence of Ru NPs had a minimal impact on τ_2_ and τ. The decreased duration of τ_2_ could be attributed to the quick transfer of electrons at the interface from the CN in an excited state to Mo_2_C. Further, Ru NPs barely affect the electron transfer rate.

To further investigate the effect of Ru-Mo_2_C on the interfacial electron migration rate in ternary catalysts, we used photoelectrochemical (PEC) detection to investigate the effect of Mo_2_C and Ru NPs on the migration rate of photogenerated electrons. The Nyquist plot of the EIS was conducted to analyze the characteristics of charge transfer resistance (Figure 6c), in which the resistance of all photocatalysts exhibited similar semicircle curves [50]. The Ru-Mo_2_C@CN has the smallest arc radius, indicating the lowest resistance to charge transfer at the interface, which aligns with its superior photocatalytic activity. Moreover, compared with CN, Mo_2_C@CN and Ru-Mo_2_C@CN samples show higher photocurrent density (Figure 6d), indicating that the introduction of Mo_2_C improved the rate of electron generation and migration. Linear sweep voltammetry (LSV) can further demonstrate the functions of Ru-Mo_2_C as a co-catalyst in the photocatalytic process. As shown in Figure S6, the Ru-Mo_2_C@CN photocatalyst exhibits enhanced cathode current and a more advantageous potential in comparison to CN and Mo_2_C@CN. This indicates that Ru NPs can enhance the surface reaction [51]. These results indicate that the Mo_2_C in Ru-Mo_2_C@CN can increase the rate of electron generation and migration, while the Ru on the surface can serve as an active site to significantly enhance the reduction effect of H^+^ in hydrogen production reactions [52].

## 3. Experimental Section

### 3.1. Materials

All reagents, including dicyandiamide (C_2_H_4_N_4_), urea (CH₄N₂O), melamine, lithium fluoride (LiF), ruthenium chloride trihydrate (RuCl_3_∙3H_2_O), molybdenum gallium carbon (Mo_2_Ga_2_C), ammonium molybdate tetrahydrate ((NH_4_)_6_Mo_7_O_24_∙4H_2_O), triethanolamine (TEOA), ethanol, hydrochloric acid (HCl), ammonia (liquid), and deionized (DI) water, are analytically pure.

### 3.2. Preparation of Photocatalysts

#### 3.2.1. Preparation of 2D g-C_3_N_4_ Nanosheets (CNs)

2D g-C_3_N_4_ nanosheets were prepared using urea as a precursor. According to the existing methods of our group [53], a ceramic crucible was filled with 25 g of urea, covered with a lid, then heated in a muffle furnace at a heating rate of 5 °C·min^−1^ until reaching 550 °C and maintained for 4 h. After cooling to room temperature, the resulting material was transferred to another ceramic crucible, heated again in the furnace at the same rate to 500 °C, and kept for 2 h. The final product, 2D g-C_3_N_4_ nanosheets, was obtained after grinding and labeling CN.

#### 3.2.2. Preparation of MXene Mo_2_C (Mo_2_C) and Ru-Doped Mo_2_C (Ru-Mo_2_C)

1 g of LiF and 0.8 g of Mo_2_GaC were added to the inner tank of the reactor, then 30 mL of an 8 M HCl solution was added to the inner tank of the reactor and stirred for 12 h. Then, heat the reactor in a 180 °C oven for 24 h. After natural cooling to room temperature, let it stand for 8 h to obtain the black solid. Wash the black solid with water and ethanol again, and centrifuge three times to obtain the bottom sediment. Finally, the bottom sediment was placed in a vacuum drying oven at 50 °C and dried for 8 h to obtain MXene Mo_2_C.

Ru-Mo_2_C was prepared in the same conditions as Mo_2_C. Differently, put 1 g of LiF, 0.8 g of Mo_2_Ga_2_C, and 40 mg of RuCl_3_·3H_2_O into the inner tank of the reactor before adding 30 mL of an 8 M hydrochloric acid solution. The remaining steps are identical to preparing Mo_2_C.

#### 3.2.3. Preparation of Bulk Mo_2_C

A total of 1.18 g of (NH_4_)_6_Mo_7_O_24_·4H_2_O was taken with 210 mg of C_2_H_4_N_4_ and fully milled. Subsequently, the mixture was transferred into a tube furnace and subjected to heating at a rate of 5 °C·min^−1^ in the N_2_ atmosphere condition until reaching a temperature of 750 °C, where it was maintained for 8 h. Following the natural cooling process to ambient temperature, thoroughly grind and calcine the product to obtain bulk Mo_2_C.

#### 3.2.4. Preparation of MXene Mo_2_C@CN, Bulk Mo_2_C@CN, and Ru-Mo_2_C@CN Composites

10 mg of different Mo_2_C species and 90 mg of CN nanosheets were dispersed in 40 mL of an aqueous solution containing 50% ethanol and stirred overnight. The solid particles were separated through centrifugation, rinsed with deionized water, and subsequently desiccated in a vacuum oven to obtain MXene Mo_2_C@CN, bulk Mo_2_C@CN, and Ru-Mo_2_C@CN, respectively.

### 3.3. Characterization and Experimentation

The characterization of samples, photocatalytic hydrogen evolution tests, and photoelectrochemical measurements are detailed in the Supplementary Materials.

## 4. Conclusions

In summary, Ru NPs were loaded onto Mxene Mo_2_C in situ for constructing ternary catalysts of Ru-Mo_2_C@CN via electrostatic self-organization for enhancing hydrogen production. The introduction of trace amounts of Ru NPs on Mo_2_C with good conductivity can significantly improve photocatalytic hydrogen production performance and effectively reduce the dependence of photocatalytic hydrogen production systems on rare and precious metals. In addition, the 2D/2D Schottky junction formed by Mo_2_C and CN, along with the presence of abundant active sites facilitated by Ru, enhances the unidirectional electronic transmission, consequently mitigating the backflow and recombination of electron-hole pairs. As expected, in the presence of a TEOA sacrificial agent, the Ru-Mo_2_C@CN catalyst can achieve a high H_2_ production rate of 1776.4 μmol·g^−1^·h^−1^ with an AQE value of 3.58% at 400 nm. These results present a new potential approach to achieve trace loading for scarce precious metals and regulate charge transport pathways.

## Data Availability

Data are contained within the article and Supplementary Materials; further inquiries can be directed to the corresponding author.

## Supplementary Materials

The following supporting information can be downloaded at: https://www.mdpi.com/article/10.3390/molecules29071684/s1. References [54,55,56,57,58,59] are cited in the Supplementary Materials.
