# Supplementary material for: Facilitated Unidirectional Electron Transmission by Ru Nano Particulars Distribution on MXene Mo2C@g-C3N4 Heterostructures for Enhanced Photocatalytic H2 Evolution
Source: Molecules. 2024 Apr 8;29(7):1684. doi: 10.3390/molecules29071684 (PMC11013833; doi:10.3390/molecules29071684)
Supplement: Supplementary file 1 [file molecules-29-01684-s001.zip › molecules-2944211-supplementary.pdf]

Supplementary Materials

Facilitated Unidirectional Electron Transmission by Ru Nano Particulars Distribution on MXene Mo_2C@g-C_3N_4 Heterostructures for Enhanced Photocatalytic H_2 Evolution

Qiuyu Chen [1,2,†], Zonghan Huang [1,2,†], Meng Liu [1,2], Xiaoping Li [1,2], Yuxuan Du [1,2], Xiaobao Chen [1,2], Dahu Ding [3], Shengjiong Yang [4], Yang Chen [1,2,*] and Rongzhi Chen [1,2,*]

[1] College of Resources and Environment, University of Chinese Academy of Sciences, Beijing 100049, China; chenqiuyu20@mails.ucas.ac.cn (Q.C.)
[2] Yanshan Earth Critical Zone and Surface Fluxes Research Station, University of Chinese Academy of Sciences, Beijing 100049, China
[3] College of Resources and Environmental Sciences, Nanjing Agricultural University, Nanjing 210095, China
[4] Key Laboratory of Environmental Engineering, Xi'an University of Architecture and Technology, No. 13, Yanta Road, Xi'an 710055, China
* Correspondence: chenyang@ucas.ac.cn (Y.C.); crz0718@ucas.ac.cn (R.C.)
† These authors contributed equally to this work.

Contents

Supplementary Material 1

1.1.Characterization of samples 1

1.2. Photocatalytic hydrogen evolution tests 1

1.3. Photoelectrochemical measurements 2

Figure S1. SEM patterns of (a)Mxene Mo_2C, (b) Mo_2C-Ru, (c) Bulk Mo_2C and (d) Mo_2C-Ru@CN. 3

Figure S2. TEM patterns of (a) Mo_2C-Ru@CN (b)~(d) Mo_2C-Ru. 4

Figure S3. High-resolution XPS spectra of Ru 3d and C 1s. 5

Figure S4. The H_2 evolution efficiency of physical mixed photocatalysts. 6

Figure S5. XRD patterns of Mo_2C-Ru@CN before and after 5 cycles. 7

Figure S6. LSV curves of CN, Mo_2C@CN, and Ru-Mo_2C@CN. 8

Table S1. Element contents in XPS 9

Table S2. Comparison of hydrogen production performance of representative Mo_2C /g-C_3N_4 reported recently. 10

Table S3. Dynamics analysis of emission decay for different samples. 12

The apparent quantum efficiency (AQE) calculation details： 13

References 14

1.1. Characterization of samples

Microstructure was analyzed using a field emission transmission electron microscope (TEM, FEI Tecnai G2 F30) and a field emission scanning electron microscope (SEM, JEOL JSM-7900F). Data on crystallinity stages were obtained by X-ray diffraction (XRD) analysis using the Bruker D8 Advance instrument at 40 kV and 40 mA with Cu Kα radiation of 0.15406 nm wavelength. With Nicolet FT-IR spectrophotometer (Nexus 470), infrared spectra were collected via Fourier transform infrared spectroscopy. Testing the surface elements and chemical conditions using the Thermo Fisher Escalab 250Xi X-ray photoelectron spectrometer (XPS). UV-VIS diffuse reflectance spectra were measured using the UV-3600 Plus spectrophotometer. Photoluminescence (PL) and Time-resolved photoluminescence (TRPL) spectra were measured using an Edinburgh Instruments FLS 1000 fluorescence spectrophotometer.

1.2. Photocatalytic hydrogen evolution tests

N2000 chromatography workstation was used to assess the efficiency of photocatalytic hydrogen production. In the experiment, 20 mg of photocatalyst were dispersed in 50 mL of aqueous solution containing 5 mL of TEOA sacrificial agent. Using a cooling water system, the reaction solution was maintained at 5°C. In order to remove any residual air from the reaction system, argon gas was introduced for 30 minutes before initiating the photoreaction. A 300 W Xenon lamp with a cut-off filter (λ>400 nm) served as the light source. H_2 was quantified using a GC-2020 gas chromatograph (Zhiheng, Shandong) with argon as the carrier gas.

1.3. Photoelectrochemical measurements

Electrochemical and photoelectrochemical measurements were conducted on an electrochemical workstation CHI-660E with a typical three-electrode cell. The electrolyte consists of an aqueous Na_2SO_4 with a concentration of 0.5 moles per liter. 4 mg of photocatalyst powder and 30 μL of perfluorosulfonic acid in 1 mL of ethanol solution were added to the base of a 4 mm platinum carbon electrode, then it was air-dried and put into the electrolyte solution as a working electrode. The electrochemical impedance spectra (EIS) recording frequency range extended from 0.02 to 1×10^5 Hz. Linear sweep voltammetry (LSV) testing using a scan rate of 0.05V/s. The photocurrent response performance of the catalyst was evaluated using an FX-300 Xe lamp with an illumination interval of 30 s. Mott-Schottky (M-S) curves were obtained in the voltage range -1 to 0.5 V with an amplitude of 0.01 V and a frequency of 200Hz.

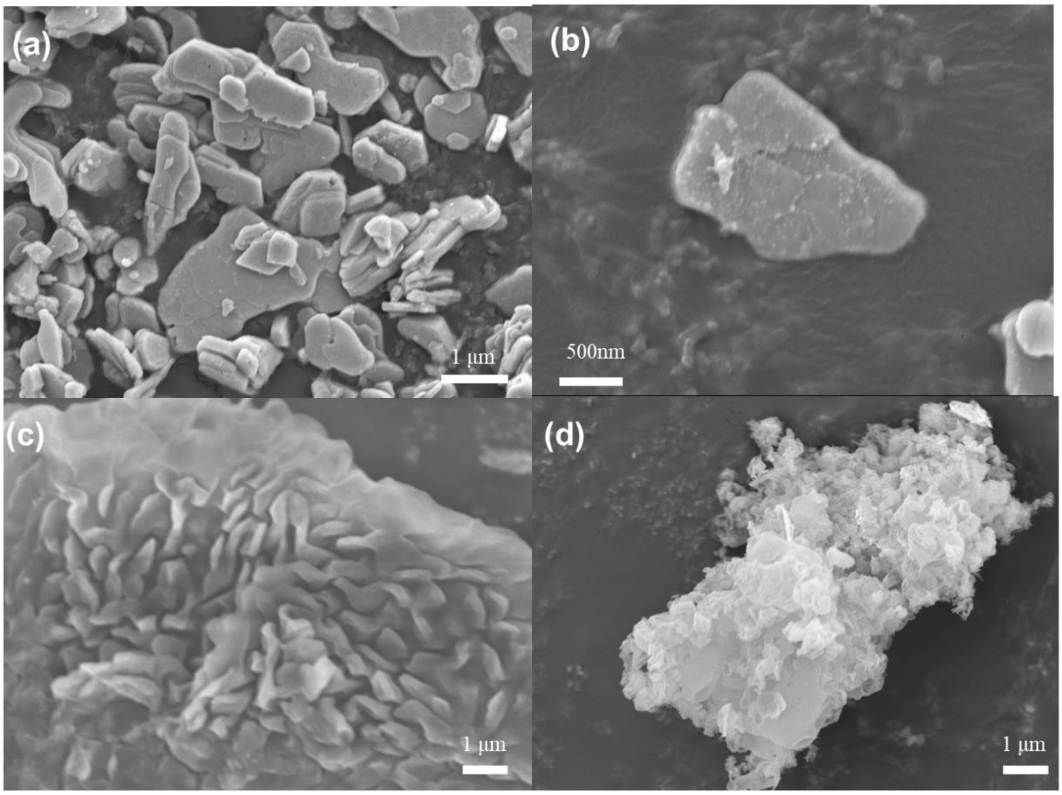

Figure S1. SEM patterns of (a)Mxene Mo_2C, (b) Mo_2C-Ru, (c) Bulk Mo_2C and (d) Mo_2C-Ru@CN.

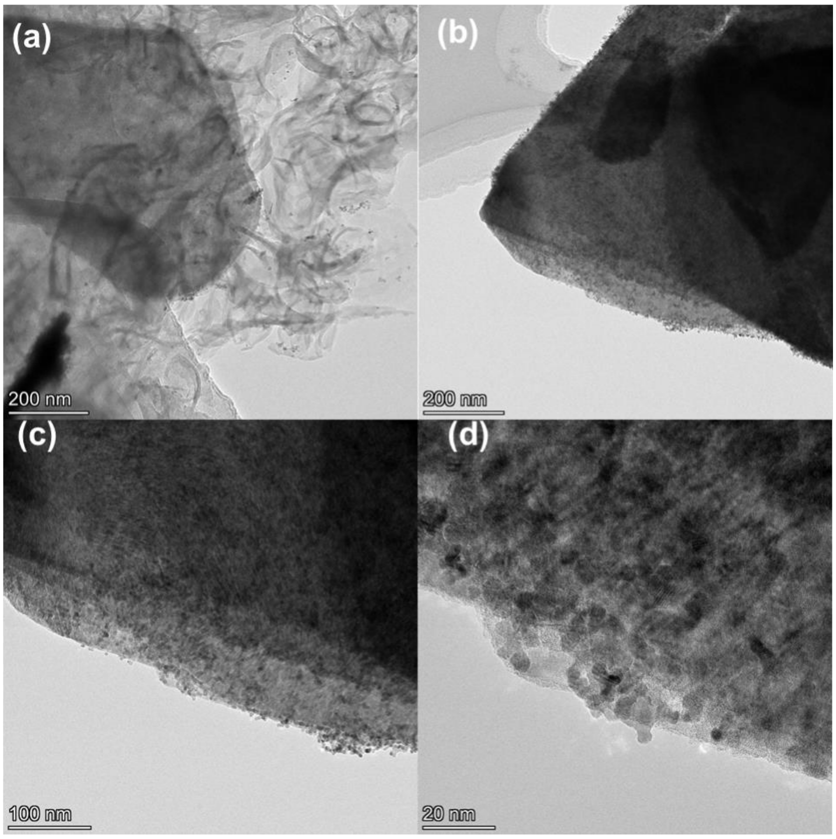

Figure S2. TEM patterns of (a) Mo_2C-Ru@CN (b)~(d) Mo_2C-Ru.

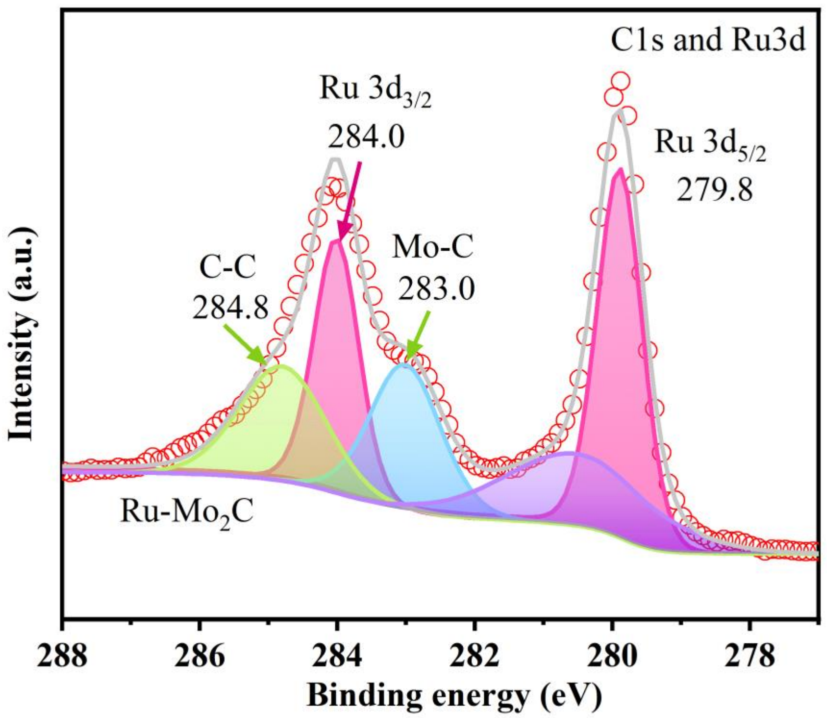

Figure S3. High-resolution XPS spectra of Ru 3d and C 1s.

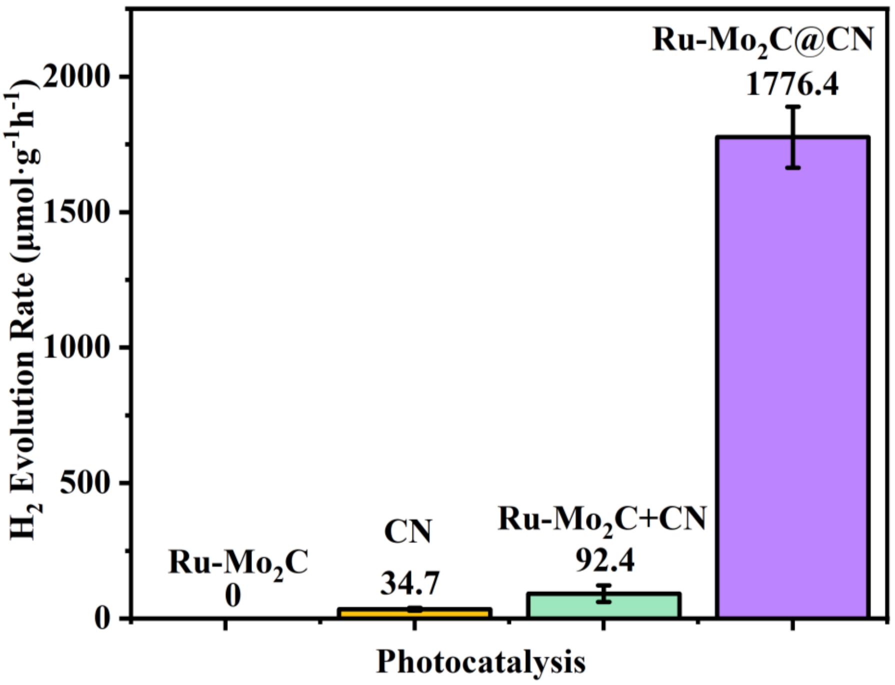

Figure S4. The H_2 evolution efficiency of physical mixed photocatalysts.

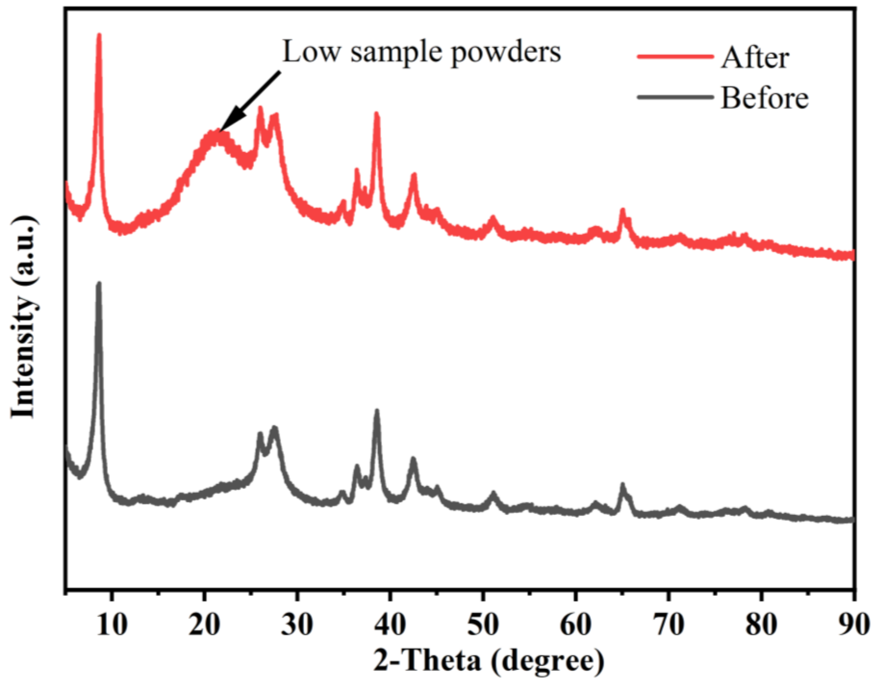

Figure S5. XRD patterns of Mo_2C-Ru@CN before and after 5 cycles.

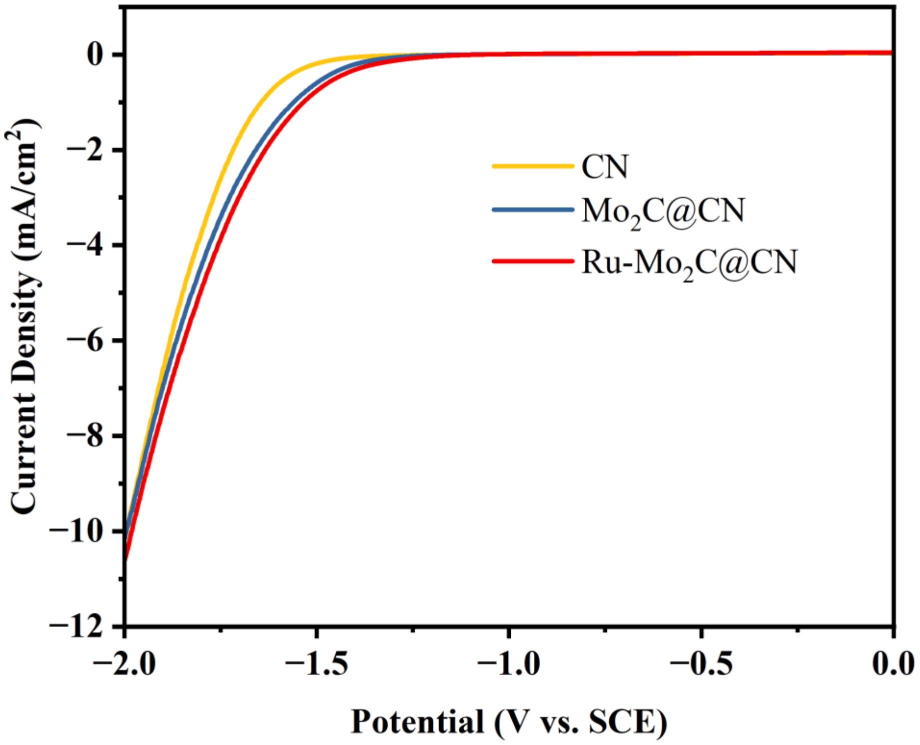

Figure S6. LSV curves of CN, Mo_2C@CN, and Ru-Mo_2C@CN.

Table S1. Element contents in XPS

Atomic %	Ru	C	N	Mo
Mo_2C-Ru@CN	0.5	44.05	53.34	2.11
Mo_2C-Ru	6.20	62.00	/	31.81
CN	52.45	/	47.55	/

Table S2. Comparison of hydrogen production performance of representative Mo_2C /g-C_3N_4 reported recently.

**Photocatalyst**	**Light source**	**H_2 production ($\mu mol \cdot g^{-1} \cdot h^{-1}$)**	**Photocatalytic conditions**	**Time per cycle (h)**	**Refe-rence**
Mo-Mo_2C/g-C_3N_4	300 W Xe lamp (λ>420 nm)	219.7	5 mg, TEOA solution	5 (5 cycles)	[1]
Mo_2C/g-C_3N_4	300 W Xe lamp (λ>420 nm)	507	50 mg, TEOA and Pt solution	3 (5 cycles)	[2]
Mo_2C/g-C_3N_4	300 W Xe lamp (λ>400 nm)	1696.4	10 mg, TEOA solution	3 (5 cycles)	[3]
Mo_2C/g-C_3N_4	300 W Xe lamp (λ>400 nm)	180	5 mg, TEOA solution	16 (1 cycles)	[4]
Mo_2C/g-C_3N_4	300 W Xe lamp (λ>420 nm)	675.27	20 mg, TEOA solution	5 (4 cycles)	[5]
Mo_2C@C/g-C_3N_4	300 W Xe lamp	2269.47	10 mg, TEOA solution	1 (5 cycles)	[6]

MoC-Mo_2C/g-C_3N_4	300 W Xe lamp (λ>420 nm)	4078	20 mg, TEOA solution	65 h (1cycles)	[7]
Mo_2C-Ru@g-C_3N_4	**300 W Xe lamp (λ>400 nm)**	1776.4	20 mg, TEOA solution	2 (5 cycles)	**This work**

Table S3. Dynamics analysis of emission decay for different samples.

Species	A_1	τ_1 (ns)	A_2	τ_2 (ns)	τ (ns)
CN	0.094	5.0858	0.009	39.7425	19.94
Mo_2C@CN	0.143	4.4783	0.010	36.6520	16.54
Mo_2C-Ru@CN	0.150	4.4262	0.010	37.3208	16.52

The apparent quantum efficiency (AQE) calculation details:

The apparent quantum efficiency (AQE) for hydrogen evolution was measured under the visible light irradiation by using a band-pass filter (λ = 400 nm) and a 300 W Xe lamp, which could be calculated as follows:

$$AQE = \frac{N_e}{N_p} \times 100\% = \frac{2 \times M \times N_A \times h \times c}{S \times P \times t \times \lambda} \times 100\%$$

where N_e is the amount of reaction electrons, N_p is the incident photons, M is the amount of H_2 molecule, N_A is the Avogadro constant, h is the Planck constant, c is the speed of light, S is the irradiation area, P is the intensity of the irradiation, t is the photoreaction time, and λ is the wavelength of the monochromatic light. The photo intensity was confirmed by Solar Power Meter (SM206). The irradiation area was controlled at 26.42 cm^2, and the photocatalytic reaction was controlled for 1 h.

When λ = 400 nm, P = 318 W $\cdot m^{-3}$, t =1 h, H_2 production = 181.2 μmol,

$$\text{AQE} = \frac{Ne}{Np} \times 100\% = \frac{2 \times 181.2 \times 10^{-6} \times 6.02 \times 10^{23} \times 6.626 \times 10^{-34} \times 3 \times 10^{8}}{26.42 \times 10^{-4} \times 3600 \times 400 \times 10^{-9} \times 318} = 3.58\%$$

References

[1] J. Dong, Y. Shi, C. Huang, Q. Wu, T. Zeng, W. Yao, A New and stable Mo-Mo_2C modified g-C_3N_4 photocatalyst for efficient visible light photocatalytic H_2 production, Applied Catalysis B: Environmental, 243 (2019) 27-35.

[2] J. Zhang, M. Wu, B. He, R. Wang, H. Wang, Y. Gong, Facile synthesis of rod-like g-C_3N_4 by decorating Mo_2C co-catalyst for enhanced visible-light photocatalytic activity, Applied Surface Science, 470 (2019) 565-572.

[3] R.-Y. Liu, L. Ding, G.-D. Yang, J.-Y. Zhang, R. Jiao, H.-Z. Sun, Hollow Mo_2C nanospheres modified B-doped g-C_3N_4 for high efficient photocatalysts, Journal of Physics D: Applied Physics, 55 (2022).

[4] J. Du, Y. Shen, F. Yang, B. Zhang, X. Jiang, C. An, J. Ye, In situconstruction of an α-Mo_2C/g-C_3N_4 Mott–Schottky heterojunction with high-speed electron transfer channel for efficient photocatalytic H_2 evolution, Inorganic Chemistry Frontiers, 10 (2023) 832-840.

[5] W. Liu, D. Zhang, R. Wang, Z. Zhang, S. Qiu, 2D/2D Interface Engineering Promotes Charge Separation of Mo_2C/g-C_3N_4 Nanojunction Photocatalysts for Efficient Photocatalytic Hydrogen Evolution, ACS Appl Mater Interfaces, 14 (2022) 31782-31791.

[6] Y. Song, K. Xia, Y. Gong, H. Chen, L. Li, J. Yi, X. She, Z. Chen, J. Wu, H. Li, H. Xu, Controllable synthesized heterostructure photocatalyst Mo_2C@C/2D g-C_3N_4: enhanced catalytic performance for hydrogen production, Dalton Transactions, 47 (2018) 14706-14712.

[7] X.Q. Tan, P. Zhang, B. Chen, A.R. Mohamed, W.J. Ong, Synergistic effect of dual phase cocatalysts: MoC-Mo_2C quantum dots anchored on g-C_3N_4 for high-stability photocatalytic hydrogen evolution, J Colloid Interface Sci, 662 (2024) 870-882.
